# Suspected twin anemia polycythemia sequence in a dichorionic, diamniotic twin pregnancy: a case report

**DOI:** 10.1186/s13256-023-03766-8

**Published:** 2023-01-29

**Authors:** Tania Jeyaseelan, Panicos Shangaris, Athina Efthymiou, Linzi Martin, Lisa Story, Surabhi Nanda, Neelam Gupta, Mudher Al-Adnani, Andreas Marnerides, Kypros H. Nicolaides, Srividhya Sankaran

**Affiliations:** 1 School of Life Course and Population Sciences, Kings College London, St Thomas’ Hospital, 10th Floor North Wing, London, SE1 7EH UK; 2 Department of Women and Children Health, Guy’s and St Thomas’ NHS Foundation Trust, London, SE1 7EH UK; 3 Department of Histopathology, St Thomas Hospital, Westminster Bridge Road, London, SE17EH UK; 4 Harris Birthright Research Centre for Fetal Medicine, King’s College Hospital, London, SE5 8BB UK; 5 Peter Gorer Department of Immunobiology, School of Immunology and Microbial Sciences, Faculty of Life Sciences and Medicine, King’s College London, London, SE1 1UL UK

**Keywords:** TAPS, Fetal therapy, Dichorionic twins, Fetal blood transfusion

## Abstract

**Background:**

Twin anemia polycythemia sequence is a rare complication in monochorionic twin pregnancy.

**Case presentation:**

We describe a case of dichorionic twin pregnancy presenting with suspected twin anemia polycythemia sequence. A 31-year-old White female, on her third pregnancy, had a routine ultrasound scan at 12 weeks gestation, which demonstrated a dichorionic twin pregnancy with one placenta located in the anterior wall and the other in the posterior wall of the uterus. At 21 weeks, a scan demonstrated a 24% growth discordance between the two fetuses with normal Doppler studies and amniotic fluid. At 27 weeks, one twin showed signs of anemia and the other polycythemia; the fetal middle cerebral artery peak systolic velocity was high in the anemic fetus and low in the polycythemic twin (1.8 and 0.5 multiples of the median). An intrauterine blood transfusion was carried out and this increased the fetal hemoglobin concentration in the anemic twin from 3.5 to 12.5 g/dL. At 29 weeks, delivery by cesarean section was carried out because of evidence from middle cerebral artery peak systolic velocity of recurrence of anemia in one twin and worsening polycythemia in the co-twin; at birth the hemoglobin concentrations were 5.6 and 24.9 g/dL, respectively. Histopathological examination confirmed dichorionicity with no communicating vessels between the two placentas.

**Conclusions:**

This is the first case of twin anemia polycythemia sequence in a dichorionic, diamniotic twin pregnancy where intrauterine blood transfusion was used to prolong the pregnancy by almost 2 weeks in a “twin anemia polycythemia sequence-like” setting.

## Background

Twin anemia polycythemia sequence (TAPS) complicates about 5% of monochorionic (MC) twin pregnancies, and is the consequence of small arteriovenous anastomoses (< 1 mm in diameter) connecting the placental vessels of the twins; an imbalance in blood flow between the fetuses results in one fetus, the donor, losing blood to the recipient so that one fetus becomes anemic and the other becomes polycythemic. Evidence for TAPS is provided from Doppler ultrasound studies demonstrating inter-twin discordance in fetal middle cerebral artery peak systolic velocity (MCA PSV) of > 0.5 multiples of the median (MoM) [1] Other signs include large echogenic placenta and cardiomegaly in the donor and congested liver in the recipient. Treatments for TAPS include expectant management, premature delivery, intrauterine transfusion, fetoscopic laser ablation of anastomoses, and selective reduction of one twin [2].

## Case presentation

This was a spontaneous conception of dichorionic (DC) twins in a healthy 31-year-old White woman with two previous uneventful pregnancies with two healthy babies. She booked her pregnancy in a tertiary hospital in London during the COVID-19 pandemic. At routine ultrasound scan at 12 weeks gestation, a DC twin pregnancy was diagnosed with one placenta located in the anterior wall and the other in the posterior wall of the uterus. There were no obvious fetal defects in either twin but there was a 16% inter-twin discordance in crown–rump length (63.9 mm versus 53.6 mm). At 21 weeks, there was a 24% inter-twin discordance in fetal size but no obvious defects in either twin, other than hyperechogenic bowel in the smaller fetus, Doppler studies demonstrated normal values in pulsatility index in the uterine artery, umbilical artery, fetal ductus venosus, and MCA. Both fetuses were male. A review at 23 weeks and 5 days showed a 26% weight discordance and bowel echogenicity in the smaller twin. Maternal blood was tested and was negative for congenital infection and cystic fibrosis.

At 27 weeks gestation, ultrasound examination demonstrated that in the larger twin there was mild ascites, cardiomegaly, enlarged echogenic placenta, and increased fetal MCA PSV to 65 cm/second (1.8 MoM). These appearances were suggestive of fetal anemia. The smaller twin, whose growth velocity was maintained on the third centile, had reduced amniotic fluid volume, normal umbilical artery Doppler, congested looking liver, and reduced MCA PSV at 17 cm/second (0.5 MoM). The inter-twin discordance in MCA PSV (1.3 MoM) raised the suspicion of possible TAPS syndrome in a DC twin pregnancy, but despite extensive ultrasound examination, there was no visible vascular communication between the two placentas. After discussion with the parents of the option of early delivery or fetal blood transfusion, it was decided to perform the latter, and blood was given to the anemic fetus by cordocentesis, which increased the hemoglobin concentration from 3.5 to 12.5 g/dL. Fetal blood sampling or exchange transfusion of the polycythemic twin was not considered at this stage.

Close monitoring every few days in the subsequent 2 weeks showed that the growth of the bigger twin was substantially reduced from the 41st to the 6th centile, and the MCA PSV gradually increased from 32 cm/second (0.9 MoM) immediately after the transfusion to 54 cm/second (1.4 MoM) at 29 weeks gestation. In the smaller twin, the growth was maintained on the third centile, but there was a gradual further reduction in MCA PSV to 14 cm/second (0.4 MoM). At 29 weeks, since both fetuses were small for gestational age and there was worsening anemia and polycythemia, respectively, it was decided to undertake delivery by cesarean section rather than carry out further antenatal interventions.

At delivery, there were no visible communicating vessels between the two placentas. One twin was pale and weighed 1140 g. He required noninvasive respiratory support and blood transfusion soon after birth due to hemoglobin of 5.6 g/dL. The co-twin was plethoric and weighed 1259 g. He was intubated and ventilated at birth and received surfactant. He had a partial volume exchange transfusion on day 1 due to hemoglobin of 24.9 g/dL. The twins had a stable neonatal course and could establish full enteral feeds. Both showed mild intraventricular hemorrhage on cranial ultrasound scan. The genotype from the samples sent from both babies was the same, confirming monozygosity.

**Fig. 1:**
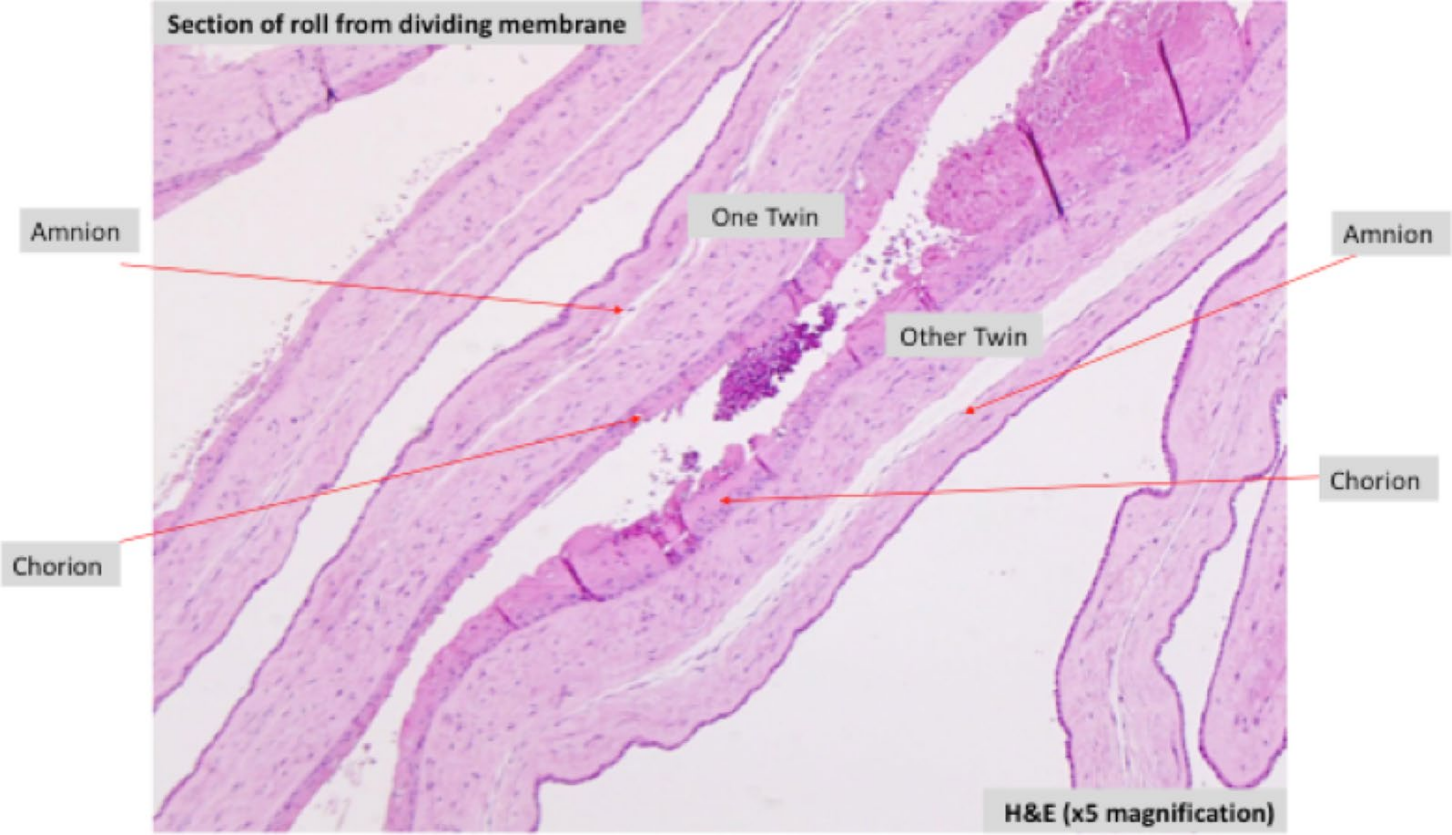
Histopathological confirmation of chorionicity on Hematoxylin and Eosin staining

Histopathological examination confirmed dichorionicity (Fig. 1), and no communicating vessels were present between the two placentas. There was an unequal partition with discs 1 and 2 contributing to 28% and 72% of the placental weight. Disc 1 showed evidence of a previous bleeding episode, and disc 2 showed villous edema. Placental sample tested negative for herpes simplex virus DNA, cytomegalovirus DNA, *Toxoplasma gondii* DNA, rubella virus RNA, and Coronavirus RNA—SARS-CoV-2.

## Discussion and conclusions

We describe a monozygotic DC twin pregnancy presenting with features of TAPS. The placental masses of the twins were distinct, with no macroscopic or microscopic evidence of vascular communication between them.

Current management options for TAPS in MC twins include expectant management, premature delivery, intrauterine transfusion, fetoscopic laser ablation of anastomoses, and selective reduction of one twin [3]. The outcome of these management options is currently under investigation in a randomized trial. In our case, the initial decision at 27 weeks gestation for intrauterine blood transfusion for the anemic twin was based on early gestational age and the desire to avoid a very premature birth. Subsequently, at 29 weeks, delivery was undertaken because of worsening growth and recurrence of fetal anemia in one twin and worsening polycythemia in the other.

There are four reported cases of TAPS that were thought to be present in DC twins. Zilliox *et al*. reported the suspicion of TAPS because of high MCA PSV in a hydropic twin and low PSV in the co-twin at 31 weeks gestation [4]. The twins were delivered by emergency cesarean section, and histopathological examination and dye test of the placentas showed several extensive arterio-venous anastomoses, as opposed to minuscule anastomosis typically seen in TAPS. Kanagaretnam *et al*. suspected TAPS in DC twins because there was inter-twin discordance in MCA-PSV of > 0.5 MoM at 32 weeks gestation [5]. After emergency delivery, placental histopathology confirmed dichorionicity; the polycythemic fetus had a darker placenta and a pale nodule of villous infarction, while the anemic fetus had a pale placenta with immature villi [5]. Tollenaar *et al*. reported a case of TAPS in DC twins diagnosed postnatally [6]. MCA PSV Dopplers were not performed antenatally due to normal growth and amniotic fluid in both fetuses; cesarean section was performed at 33 weeks due to fetal distress following uterine contractions, and there was a 12.6 g/dL discrepancy in hemoglobin concentration between the twins. Histopathological analysis of the placenta demonstrated veno-venous anastomosis. In this case, acute peripartum TTTS was also considered a possible diagnosis, where blood shift between twins occurs rapidly, often triggered by uterine contractions in labor. Lee *et al*. also reported a case of TAPS diagnosed postnatally in DC twins who were delivered by emergency cesarean section following premature rupture of membranes at 33 weeks [7]. Hemoglobin concentration discordance was 14.4 g/dL, and the authors suggested that there was acute peripartum TTTS. The severe consequences of TAPS, such as polycythemia leading to limb ischemia and severe cerebral damage, should not be underestimated [3, 6].

This is the first case of a DC twin pregnancy with no vascular communications between the two placentas, with the fetuses demonstrating “TAPS-like” features, requiring intrauterine blood transfusion to prolong the pregnancy.

## Data Availability

It is possible to obtain access to further anonymised histopathological and ultrasound images through the corresponding author.

## Abbreviations

TAPS: Twin anemia polycythemia sequence
MC: Monochorionic twin
MCA PSV: Middle cerebral artery peak systolic velocity
DCDA: Dichorionic, diamniotic
MoM: Multiples of the median
